# The perspectives of educators, regulators and funders of massage therapy on the state of the profession in British Columbia, Canada

**DOI:** 10.1186/2045-709X-21-2

**Published:** 2013-01-07

**Authors:** Farah M Shroff, Inderjeet S Sahota

**Affiliations:** 1 Department of Family Practice and School of Population and Public Health, University of British Columbia, Third Floor David Strangway Bldg, 5950 University Blvd, Vancouver, BC, V6T 1Z3, Canada; 2 Department of Biomedical Physiology and Kinesiology, Simon Fraser University, Simon Fraser, Canada

**Keywords:** Health care professionals, Professional education, Alternative and complementary health care, Massage therapy

## Abstract

**Background:**

Registered Massage Therapists (RMTs) are valuable members of the healthcare team who assist in health promotion, disease prevention, treatment, rehabilitation and palliation. RMT visits have increased across Canada over the past decade with the highest increase in British Columbia (BC). Currently, RMTs are private practitioners of healthcare operating within a largely publicly funded system, positioning them outside of the dominant system of healthcare and making them an important case study in private healthcare. In another paper we examined the perspectives of RMTs themselves. Here, we offer perspectives of regulators, educators and funders of Massage Therapy (MT) on advancement of the profession.

**Methods:**

We interviewed 28 stakeholders of MT in BC – including members of the MT regulatory board, representatives from MT colleges in BC and public and private health insurers.

**Results:**

All three groups identified research, particularly on efficacy of MT, as playing a vital role in enhancing the professional credibility of MT. However, participants noted that presently research is not a large feature of the current MT curricula and we analyze why this may be and how it can improve. Finally, conferral of baccalaureate degree status could assist RMTs in gaining recognition with the general public and other healthcare professionals.

**Conclusion:**

RMTs have potential to ameliorate population health in a cost-effective manner. Their role in British Columbia’s healthcare landscape could be expanded if they produce more research and earn degree status.

## Background

Registered Massage Therapists (RMTs) in British Columbia (BC) are autonomous, self-regulating professionals. The majority of their practices operate as privately funded businesses providing mainly musculoskeletal healthcare services [1]. The majority of their funding comes from public and private insurance companies and their educational institutions are also for-profit businesses [2].

Massage Therapy (MT) regulation varies across Canada. Only BC, Ontario and Newfoundland currently regulate the practice of MT [3]. RMTs in BC must register with the College of Massage Therapists of British Columbia (CMTBC). There are three levels of legislation governing MT in the province: 1) the Health Professions Act of BC, 2) The Massage Therapists Regulation and 3) the By laws of the College of Massage Therapists in BC [4]. In BC, membership requires a minimum of 3000 hours, or approximately three years of training, making the approximately 2600 RMTs [5] in BC some of the best trained massage therapists in the world [6]. With cutbacks to provincial healthcare coverage, limited MT treatments are available for low income individuals [7]. Those who seek MT services due to motor vehicle or workplace accidents also receive some, albeit minimal, coverage through government administered insurance agencies with a physician’s referral [7]. Presently, most RMTs’ clients have coverage through privately funded extended health plans. Other patients pay for MT out of pocket. Therefore, RMTs are typically providers of private healthcare within BC. In recent years, the CMTBC has been lobbying the provincial government to reincorporate MT as a publicly insured service, citing cost-effectiveness and increased efficacy in the use of MT for the treatment of soft-tissue and other musculoskeletal disorders [8]. Many RMTs, on the other hand, feel they were inadequately compensated under government funded insurance and are not interested in returning to the previous funding scheme.

MT, physiotherapy and chiropractic have overlapping scopes of practice; however, compared to these other two professional groups, RMTs experience a number of difficulties. The general public and other healthcare providers often misunderstand RMTs. Conflation with sex-trade workers is a threat to the professional reputation of RMTs [9]. Furthermore, they face competition from unregulated bodyworkers who claim to do the same work, particularly relaxation and stress reduction massage. Touch therapies are generally misunderstood in Canada; in other countries, such as Thailand and India, massage is a time-honoured tradition understood by the majority of the population as beneficial for health [10].

Canadian RMTs typically have a relatively short career span due to the physically demanding nature of their work [2]. Moreover, RMTs depend on referrals from Medical Doctors (MDs) for clientele. Without a note from an MD, most RMTs’ clients would not receive insurance coverage [2,7]. Finally, RMTs’ are almost completely dependent on private insurers for their livelihood and while many RMTs feel they are sufficiently compensated, tensions between RMTs and private health insurers exist. These complexities render the study of MT important because they provide a popular service currently outside the realm of publicly-funded healthcare for most people. We asked the question: how can the MT profession advance given these constraints? In examining these issues we illustrate various challenges faced by RMTs as privately funded healthcare providers.

In this study we interviewed various stakeholders who were involved in the current practice of MT in BC: colleges of MT, regulators of MT and private and public healthcare insurers. In investigating avenues for greater success for this profession we learned about market-forces at play within MT; RMT education and practice are mainly privately funded so this profession relies to a great extent on financial backing from business people. In Canada, since the early 1960’s many healthcare professionals are publicly funded; this includes physicians, nurses and other professionals [11]. The spiralling costs of drugs, hospital administration and doctor’s earnings, among other factors, threaten the financial sustainability of the Canadian public healthcare system [12]. While approximately 30% of healthcare [13] services are currently contracted to private companies, this percentage will likely increase in the future. It is, therefore, insightful to explore the contours of privatized healthcare as it currently exists in Canada.

### Purpose

In assessing how this profession could move forward as a privately-funded system we sought to gain a comprehensive perspective on MT within the province. In another paper [2] we summarized the perspectives of RMTs in regards to their own profession. The purpose of this paper is to examine the perspectives of other stakeholders- MT educational institutions, regulators and funders – soliciting their views on the advancement of this profession.

## Methods

### Recruitment

The research team compiled a list of all stakeholders in MT and contacted them via phone and email. All stakeholders who responded to our requests were keen to be involved. While the research team attempted to contact both elected officials as well as staff within the provincial government we received no response. We were, therefore, unable to include the voice of government in this study. Participants were not financially compensated for their participation.

### Protocol

Questions were presented to study participants in written form and are appended here (Additional file 1:Appendix). Each participant signed a consent form. Ethical approval was granted by the University of British Columbia Behavioural Research Ethics Board. We employed self-completed questionnaires, focus groups and one-on-one interviews. The first author of this paper conducted all interviews and focus groups. All responses were digitally recorded and partially transcribed in order to capture participants’ exact words. We employed qualitative methods of questionnaire design, interviewing and analysis.

### Participants

Ten members of the organization responsible for regulating the practice of MT within the province, the MT regulatory body (MTRB) were interviewed. We distributed questionnaires in advance and collected responses during the focus group. We interviewed twelve administrators representing all MT colleges within the province. All interviews were conducted at the college campuses, except one which was interviewed via email.

We interviewed members of one private and two public insurance companies. Where possible, we held interviews with insurance company representatives in charge of MT claims. We conducted focus groups with four staff members of a private insurance company. We thus interviewed six representatives from public and private insurance companies across the province.

In total, 28 people participated in this study.

### Data analysis

Members of our research team partially transcribed all interviews and answers to each question were inserted into appropriate sections. For example, we had section headings of “Do you believe research can advance the profession of massage therapy?”, “Are you satisfied with the current regulation of MT research curricula in schools?” and “In your opinion, what aspects of MT require further research?”. From the categorized sections, we analyzed responses using qualitative techniques of coding, diagramming and thematic analysis. In addition to audio-recording, the principal investigator took notes throughout the interviews. Interviews lasted from 30 to 90 minutes.

## Results & discussion

Although many ideas were presented, almost all stakeholders felt that increased research on the benefits of MT would help elevate the profession. Stakeholders’ views on advancing the profession are summarized below. As they identified research as a particularly important facet in moving MT forward, we discuss it in more detail.

### Advancing the profession: regulators and educators

Members of the MTRB felt that acceptance from other health professionals, respect from government as well as strong validation of what RMTs do through research would advance the profession.

"“[RMTs would benefit from] strong validation of what we do through research [in addition to] greater acceptance by other health professions, greater referrals [and] no GST.”"

"“If we had national professional recognition we’d be able to move forward.”"

"“[We need to] integrate evidence into practice.”"

"“[We would benefit from respect from] other health professionals, respect from government.”"

The response of MT colleges to “what steps do RMTs need to take in order to elevate the status of the profession” included the following: increasing research in the area of MT and health and educating the public on the qualifications and roles of RMTs.

"“People don’t know how well educated RMTs are. RMTs have excellent training – let’s talk to the public!”"

"“[We need to] establish evidence-based research.”"

One MT college administrator felt that providing leadership to spearhead the needs of RMTs as well as establishing best business practice courses in colleges could help RMTs succeed in their careers.

"“Resistance to business practices in colleges. Chiros realised this and train to be both a good business person and practitioner. The MTA (Massage Therapists’ Association) could look at AMTA (American Massage Therapy Association) and invite them to speak at conferences – they know how to help members.”"

"“Leadership! We need better leadership to raise [the] status of our profession. Let’s bring in the expertise to help us move forward. It would be good to have leaders setting direction for the program [but] we need all the stakeholders working together!”"

Massage colleges and the MTRB recognized the significant role of research in getting greater acceptance for MT from other healthcare professionals and respect from the government. All stakeholders highlighted the importance of research in moving the profession forward. However, although the importance of research was frequently cited, peer-reviewed literature and research education practices are minimal. This topic is discussed in more detail next.

### Role of research in moving MT forward

#### The importance of research in advancing the profession

The MTRB was interested in research on safety and efficacy of MT as well as cost-effectiveness. Research on safety of MT was cited by 6 of the MTRB respondents as an important component of MT that requires further research. The primary role of regulatory bodies is to protect the public. It is therefore understandable that safety is a top concern for regulators. However, there is currently no documented evidence of side effects of MT [14]. It is, therefore, considered to be safe.

“[Research is important] to help produce evidence or risk of harm in order to communicate more effectively with the government. It would be helpful in our negotiations with the Ministry of Health.”

Research on efficacy was cited as important by 3 MTRB respondents and the remaining one thought cost-effectiveness would be the most valuable aspect of MT to research. Interestingly, if responses were analyzed by summing the total of the top two responses to the question, “what aspect of MT should be researched further,” 9 MTRB respondents cited efficacy. This highlights the importance of researching efficacy of MT; an issue echoed in later statements by members of public and private insurers. Understandably, people want to prove that MT improves health status; therefore, research on efficacy is essential to the advancement of this profession.

"“If we want the profession to grow and advance, [research] has to be very important.”"

"“We can’t expect physios and others to view us (RMTs) as their peers when we don’t do research.”"

All participants representing MT colleges indicated that they believe research is very important to the profession of MT. One MT college administrator noted:

"“If we want the profession to grow and advance, research has to be very important. If we’re saying our training and our practice in the health field is equal to others we have to be doing it too. For RMTs to take it to the next step they’d need a researcher to work with.”"

Both MT colleges and the MTRB identified research as an important component in advancing the profession. For many, research proving the efficacy and safety of MT would be beneficial in enhancing the profession’s image in the eyes of the provincial government. More research was also cited as important in gaining recognition from their peers, such as physiotherapists, chiropractors and importantly, medical doctors, as valuable members of the healthcare team.

#### Research education in MT colleges

Responses of MTRB participants with the current regulation of research education in MT colleges varied from somewhat satisfied to extremely unsatisfied. Most MTRB participants (8) were somewhat satisfied with the current regulation of MT research curricula in MT colleges whereas 1 was extremely unsatisfied and 1 was undecided.

"“[It’s a] work in progress. Faculty needs to buy into research with their world view.”"

"“Extremely unsatisfied [with the current regulation of MT curricula]; further integration is needed.”"

MT colleges devote very little time in their curriculum, approximately 30–40 hours of the 3,000 hours, to teaching research methodologies. Some conduct a lecture-based research and statistics course which focuses on research literacy, validity and research design. Typically the research training consists of a single course taken in the advanced years of the program. However, many MT schools report that research training courses are not well received by the students. One college participant reported that 80% of students ask “why do we have to take this course?” Others reported that those with degrees tend to be more interested in research while other students could see the value of being able to search the literature.

MT colleges provided contradictory reflections regarding the status of their students’ research capabilities. Participants reported that their students had varying levels of research literacy, with some students studying the literature regularly while others rarely doing so and lacking critical thinking skills. In contrast, half the college participants stated that the majority of their graduates could design and conduct independent research. However, this sentiment was not echoed in statements given made by MT college participants. For example, one college representative stated that students do not have the experience or the time (while in college) to participate in research. Their college had only one student in a class of 33 that actually participated in their study last summer.

It is unlikely that a substantial portion of MT college graduates could indeed design and conduct independent research for a number of reasons. Firstly, courses regarding research design and statistical analyses form only a small percentage of the total courses taken during MT college training: approximately 30–40 of the total 3000 hours. This is largely due to MT colleges’ focus on accrediting their graduates to practice under the MTRB regulations. Secondly, as noted above, RMT students do not seem keen on conducting research; instead, their focus is on clinical practice. The reason they choose to enter MT is to work with patients and MT research is often less lucrative than clinical practice [2]. Also, some administrators noted that most of their faculty do not have a research background and, therefore, are not willing or able to implement new research courses into the curricula. If faculty are not able to teach MT students about research design and/or literacy skills it is unlikely that many graduates could conduct MT research projects themselves. For half of the MT college participants to state, therefore, that their graduates could design and conduct independent research is questionable.

#### Barriers to incorporating research education in MT colleges

Participants noted that the greatest barriers to MT research in colleges were time, money and lack of research expertise. One MT college board member summarized these barriers:

"“One, our curriculum [is] designed to be a clinical model. My job is to prepare students to pass board exams. They have 30–40 hours out of 3000 hours to spend on research. I have a 5 year plan which includes research. Two, the majority of our faculty are not well versed on how to [conduct] research. Three, time. Four, our goal is to get accredited [by the provincial MTRB]; therefore, our main goal is accreditation.”"

To overcome these barriers, college representatives said that it would be helpful if someone was designated at the colleges or other MT professional associations to review the literature, organize new research by category and then convey summaries of the information to the staff and members. Another representative said that RMTs and researchers should work together: therapists could form partnerships with people who are knowledgeable in research to grow research competency and increase the faculty’s interest. However, all felt that the college curricula were already so full that it would be difficult to incorporate another module on advancing research capacity.

The MTRB participants believed that encouraging members to enroll in research-based courses for continuing education credits (CEC) is an important step in improving research literacy within the RMT community. Other ideas included increasing the amount of information provided on MT-related websites and making research literacy a mandate for MT colleges.

All the colleges were interested in collaborating with organizations such as the MTRB or other MT professional associations to develop, test and evaluate a best practice guideline to mentor students to carry out research. However, one wasn’t sure how it would work or fit into the current curriculum. Another stated that the medical model does not appeal to them and that they would prefer a broader model that would encompass all facets of MT including the wellness and holistic aspects. One college representative said that they would prefer to work with the MT professional organizations to benefit all RMTs, not just students. From their experience, they were better off using just a few well-trained RMTs for studies.

When interviewing the MTRB, the majority of respondents (9) were also interested in pursuing cooperative research projects with other MT-related organizations. They emphasized that their organization would likely play a funding-provider role as conducting research was not a mandate of the organization.

All the colleges needed funding to support their ability to integrate the research competencies throughout the curriculum. This would have to be mandated by the MTRB and supported by them, said one college. Ultimately it was felt that the board examination process drove everything. This is echoed in a statement from one MT college representative below:

"“In the end, the issues are time, money and motivation. Ultimately the board examination process drives everything. If the board examiners don’t incorporate research into the process, the motivation isn’t there.”"

Other college representatives felt that there were too many who would be resistant to such a move. As all MT colleges felt that the current curriculum is tight, creating space for the development of research literacy means removing other courses which they are unwilling or unable to do.

"“We need a curriculum review. Do we need 4000 hours or do we pull out stuff to make space for research? We can’t lose manual skills [and] the university recognizes what we are doing [but] what will it take [for us] to become a degree-granting institution?” "

In general, the most significant barriers to incorporating research training in MT colleges were time and the focus of the MTRB board examination. Both MT colleges and the MTRB stated they were keen on collaborating with each other and with other MT-related organizations to increase the quantity and quality of research currently being conducted in the field.

### Role of research in the advancement of other professions

Increasing the number and quality of studies examining the efficacy and safety of physiotherapy, nursing and chiropractic practice have helped these professions gain recognition from both other healthcare professionals and governing bodies of healthcare. RMTs and stakeholders of MT feel these issues are of great importance in advancing the profession of MT within British Columbia [2]. Chiropractic, physiotherapy and nursing have advanced partly through research. Each of these professions emphasizes research literacy and other research skills in their curricula. A significant number of their practitioners are actively engaged in evidence-based studies producing a number of robust research articles annually. This has been discussed in detail previously [15].

### Why is evidence-based MT lagging behind?

Both RMTs [2] and other stakeholders of MT recognize the importance of high quality research in advancing MT’s professional recognition with other healthcare professionals, governmental healthcare organizations as well as the general public. Lack of research capacity is a substantial obstacle to moving MT forward. There is a lack of research training in MT colleges and consequently, a lack of research personnel. Ideally, academic researchers and RMTs ought to be working in teams in order to conduct studies on the effects of MT. Part of this problem stems from the fact that research skills and literacy are not emphasized in MT colleges. The focus of these colleges is to have their graduates become accredited RMTs by the MTRB which means passing the MTRB board examinations. As this is the final goal for most MT colleges and as the examination process is focused on clinical knowledge and skills and less on research, there is little motivation to pursue research literacy and development.

Another reason has to do with the limited funding opportunities available to RMTs who wish to involve themselves in MT research. Only two organizations across Canada have directly provided funding for MT research: the Holistic Health Research Foundation of Canada and the Massage Therapy Foundation. The limited funding opportunities, coupled with the fact that clinical MT practice is more lucrative and MT college gradudates are often saddled with large debts means the incentive to pursue MT research is low.

### Degree status for MT education could help advance the profession

Some MTRB participants liked the idea of RMTs earning a degree from a public university and others did not. Half of respondents were supportive of the idea, 3 respondents opposed the idea and 2 were undecided. Of those that were supportive, maintaining standards on par with other health professionals, increasing opportunities to transfer course credits and recognition for the amount of course work completed in the MT program were frequently cited reasons.

"“University degree status is unavoidable. We could create a working agreement between colleges and universities to make [the] TRU (Thompson Rivers University) agreement mandatory. Education may be better delivered at a public university than a private college [as we] don’t need to raise the bar to get into university.”"

"“Yes – to remain viable as profession.”"

"“Yes … in order to remain competitive with other health professions.”"

"“Last time we had a stakeholders’ meeting people were not keen to mandate a degree, to allow laddering is good. I’d be quite upset if this happened. In Ontario there is public education and it has created chaos.”"

In meeting with colleges of MT they too noted that they would like to see RMTs gaining a university degree but they would like to be granted the authority to grant degrees themselves. One of the college’s faculty members suggested that 3,000 hours of education was more than an undergraduate degree so it was only fair that RMTs should earn a degree and not a diploma.

"“What will it take [for us] to become a degree-granting institution? [Our graduates require] 3000 hours of training, [that is] more than university degree.”"

"“[We would like a] degree program [but] universities may not because private colleges [would be] offering it.”"

"“A local university/college was willing to partner with us [to obtain degree granting status for RMTs] but it didn’t work after 3.5 years of trying. We spent approximately $100,000 trying to obtain such a status.”"

Baccalaureate status has helped advance the professions of physiotherapy and chiropractic, professions with similar “hands-on” approaches to healthcare [16]. To become a chiropractor in Canada, for example, one must complete undergraduate credits and then enrol in a postgraduate chiropractic college for 3–4 years [17]. Physiotherapists undergo a similar process with most students completing an undergraduate degree and then enrolling in a Masters of Physical Therapy program now available in many public universities across the country [18]. Increased educational requirements for both chiropractors and physiotherapists have meant, 1) graduates of these programs are more knowledgeable regarding clinical and research practice in their professions and, 2) exclusivity related to requiring a post-baccalaureate degree allows colleges to be highly selective in selecting their students for these programs, accepting only the most qualified and/or accomplished [16]. It is likely expected that a push for obtaining degree status for RMTs could facilitate their advancement in a similar way.

### Insurance company participants express requirements for evidence-based practice

The top health issues that our insurance company participants address are mental health issues, as well as soft tissue injuries – neck, back, shoulders and knees. All company representatives noted that RMTs effectively address these health issues and that MT provides positive health benefits. They also believed that MT successfully prevents disease and treats injury although some wanted more research-backed evidence regarding the effect on disease prevention. In terms of helping people get back to work, one insurer felt that RMT outcomes were good but not great, as the return to work (RTW) outcomes during 2000–2006 was, on average, 44.6%. The goal was to improve the RTW within 5 weeks of first visit to 50%.

Insurers also told us that their company would cover RMTs more widely for visits and larger maximum annual pay outs if there was proof of effectiveness, research and more endorsement from MDs. One said that they always gave approval to MT if doctors had recommended it. A review of massage therapy by a BC physician recommended that the physician who has referred a patient should ask the therapist to provide a progress note describing *measured* outcomes of the therapy [19].

One insurer felt that the perception of MT as a ‘touchy/feely therapy’ had to change and that MT needs to be seen as an *active therapy* that shouldn’t create dependency. The concept of active therapy came up in all interviews with insurance companies as they continuously compared exercises taught by physiotherapists to time on the table with RMTs. They encouraged the MTRB to make MT as ‘active’ as possible.

Empirical evidence linking MT to benefit and outcomes would make RMT more appealing to all insurance company respondents we interviewed. All the insurance companies stated that they utilized research literature in determining the approval of claims and one even had an evidence-based practice group which did reviews of findings. All wanted to receive research literature on MT including information on return to work outcomes, new studies, emerging techniques and other focused information.

Concurring with the views expressed by the insurance companies, one member of an MT college stated:

"“The results speak for themselves. RMTs that do not keep up with best practice techniques may not get the results in their practice. Insurance companies see the poor results (and poor reporting of outcomes) by some therapists and generalize to all massage therapists. Published studies in peer-reviewed journals are what the insurance providers want to see. Even case reports and case series can be of benefit to the profession.”"

The MT-related organizations are keen to improve relations between RMTs and insurance providers. They hold information sessions for RMTs to learn about interacting with insurance companies effectively.

### Abuse of claims

Another important issue insurance company participant’s noted is the perceived abuse of claims, although they noted that these were not necessarily done deliberately. One private insurance company was concerned that some RMTs are billing for medically unnecessary MT. We repeatedly heard one example during an interview: a $300 receipt from a spa from an RMT. When the insurance company asked for an itemized receipt they saw that $300 included wine, chocolate covered strawberries and other items that were not legitimate. This company urged RMTs to be better informed about billing only for medically necessary MT. Currently, most RMTs use generic, non-specific receipts. They suggested that if RMTs had more official receipts they would be better able to judge the legitimacy of the claims. The other concern this private insurer had was that MT claims had substantially increased over the past 2 years. Participants were disturbed by this trend as they suspected part of the increase was due to illegitimate expenses.

Insurer respondents felt that there needed to be a clear distinction between medical massage and wellness massage. Within the MT profession there is a parallel discussion where some RMTs would like the profession to devote itself to rehabilitation and purely therapeutic practices whereas others believe MT better serves population health by being more comprehensive as a form of health promotion, disease prevention, rehabilitation and palliation. The latter group incorporate wellness massage into their practice. A third group, the spa practitioners, only practice relaxation massage. Insurers made it clear that they were interested in funding medical massage.

However, it was interesting to ask each participant about their own personal experience with MT. Those who had received regular MT stated that they were willing to pay out of pocket for relaxation massage and be covered for injuries only. The one participant who had never received the services of an RMT held the most negative views on MT. This suggests that personal experience with MT can have a significant influence on individuals’ professional views of it. Literature on holistic practices in general echo this idea. For example, physicians who have received MT treatment for their own healthcare services or have more knowledge regarding MT feel more comfortable discussing MT treatments with their patients [20].

### Stakeholder dynamics

Each of these stakeholders has an agenda. The regulatory agency’s agenda is related to their mandate of protecting the public. The agenda of the insurance companies is to protect their profits while assisting patients to improve their health. The agenda of the educational institutions is educating RMTs while maintaining profitability. Profit margins are important to at least two out of our three stakeholder groups and this is where conflict within the profession may exist. Furthermore, within each of these groups also lie many conflicts, partly based on personalities, egos and politics. The profession of MT in BC is fraught with internal struggles that impede its progress. It will not move forward until the agenda of all stakeholder groups are aligned and unity amongst groups and individuals is achieved.

## Conclusion

This paper examined various factors in the development and enhancement of the profession of MT. Stakeholders of MT in British Columbia, Canada, identify research as a particularly important contributor. All stakeholders agree that more research would enhance the profession. Regulators specifically wanted more research on safety although no evidence currently exists to prove MT has serious adverse side effects. This emphasis on safety is understandable given that regulators’ main task is to protect the public. Other stakeholders, including RMTs themselves, are more interested in MT research that examines the efficacy of MT treatments.

Many MTRB participants were not satisfied with current curricula offered in MT colleges regarding research skills. Given the limited research training currently offered in MT colleges, many graduates of MT colleges are unable or unwilling to pursue MT research as a career option over or in combination with clinical practice. This is further exacerbated by the fact that limited funding opportunities for MT research exist across the country and since many RMTs graduate from college with large debts, engaging in clinical practice which is more profitable is often the priority. MT organizations, including colleges and the MTRB, are keen on collaborating with each other to increase the amount of MT research conducted.

Educators clearly stated that they saw research as an important way of advancing the profession but identified four main barriers: 1) faculty do not have the skills to teach research, 2) students are not keen to pursue research as a significant part of their studies, 3) there is very little room in a tight curriculum to incorporate research, 4) the MTRB board examination drives the current curriculum and research is not an important component of the board exam. Time, finances and motivation are additional barriers.

All stakeholders agreed that MT would move forward were their practitioners’ education validated by the conferral of baccalaureate status. Chiropractors and physiotherapists, professions with similar scopes of practice as RMTs, currently earn not only an undergraduate degree but postgraduate degrees which appear to have served them well. Currently MT colleges offer a 3,000 hour program which is more time than a standard university-level bachelor’s degree. If RMT colleges earn degree granting status, adding research to the curriculum will be a condition of this transition. There are two avenues to move toward this goal. The first would be to receive training entirely through a publicly funded university. Currently in British Columbia, trainees in MT at a private college can transfer three years of college credits to a public university towards a degree. However, no degree programs for MT exist and, therefore, individuals interested in pursuing a degree must study at private colleges beforehand. If students attend a public educational institution they are eligible for more loans and scholarships so they could potentially graduate with less debt. The second option would be for privately funded colleges to grant degrees themselves.

In addition to baccalaureate status, research has greatly impacted the advancement of nursing and other “hands-on” therapies such as physiotherapy and chiropractic. The combination of baccalaureate degree status and increased research into the efficacy of nursing, physiotherapy and chiropractic has increased the recognition of these professions as valuable components of healthcare by both their peers and various governments. A similar push is required, and is indeed occurring, in the profession of MT. However, obtaining undergraduate degree status for RMTs’ education has in itself been difficult, as indicated by some MT colleges actively engaged in the process.

MT is not understood by other healthcare professionals and there is an “identity crisis” within the profession. On the one hand, private funders interested primarily in profits, are willing to engage in the commercialization of MT. For example, connecting MT to aesthetic and cosmetic services and other beauty or fashion-oriented businesses is one way of increasing profits in this field. Indeed, one of the MT colleges we interviewed engages in such practices. Another “identity crisis” exists between MT as a medical or as a wellness profession. Insurance companies are keen to fund only medical MT and many RMTs are proponents of MT as a treatment-oriented medical practice. Others believe that MT ought to cover the complete spectrum of healthcare, incorporating wellness, disease prevention and rehabilitation. Agreement on the scope of MT practice has not been reached within or outside the profession. Further adding to this divide is the lack of unity between stakeholders of MT in the province. Acrimony and animosity between stakeholders characterize many relationships in BC’s MT community. These divisions in the MT community have created a lack of leadership and common direction within the profession. The skeptical positions of other healthcare providers, moreover, and governments towards MT create significant barriers to the advancement of this profession.

Besides the internal and external forces that prevent the advancement of this profession is the serious concern, on the part of insurers, that abusive claims occur. Insurers repeatedly noted that illegitimate charges were being made by RMTs.

In this paper we examined various issues related to the advancement of MT. MT is primarily a private healthcare service provided by practitioners who are largely outside the provincial healthcare system and it is misunderstood by other healthcare professionals. Despite these difficulties, British Columbians are increasingly accessing the services of RMTs and, therefore, the demand for RMTs has increased. Future research in this area should examine economic forces at play in the medical marketplace of BC. A study examining the financial and economic environment within which RMTs practice would be helpful to shed light on the larger context. When the profession is able to deal with the internal and external challenges we discuss here it will be able to move forward and be recognized as a significant player in healthcare in the province.

## Abbreviations

AMTA: American Massage Therapy Association; BC: British Columbia; MD: Medical Doctor; MT: Massage Therapy; MTA: Massage Therapists’ Association; MTRB: Massage Therapy Regulatory Board; RMT: Registered Massage Therapist.

## Competing interests

The author(s) declared no potential conflicts of interest with respect to the research, authorship, and/or publication of this article.

## Authors’ contributions

FMS designed and conducted the study. FMS and ISS analyzed and interpreted the data and prepared the manuscript for publication. Both authors read and approved the final manuscript.

## Authors’ information

Farah M Shroff, PhD, is an Adjunct Professor in the Faculty of Medicine at the University of British Columbia in Vancouver, Canada. Inderjeet S Sahota, MSc, is a Research Associate in the Department of Biomedical Physiology and Kinesiology at Simon Fraser University in Vancouver, Canada.

## Supplementary Material

Additional file 1: Appendix MTRB Questionnaire. Insurance Company Questionnaire. Massage Therapy Schools Questionnaire. Click here for file
