# Supplementary material for: The perspectives of educators, regulators and funders of massage therapy on the state of the profession in British Columbia, Canada
Source: Chiropr Man Therap. 2013 Jan 7;21:2. doi: 10.1186/2045-709X-21-2 (PMC3565927; doi:10.1186/2045-709X-21-2)
Supplement: Additional file 1: Appendix — MTRB Questionnaire. Insurance Company Questionnaire. Massage Therapy Schools Questionnaire. [file 2045-709X-21-2-S1.docx]

# Appendix

## MTRB Questionnaire

(1) Do you believe that research can help advance the profession of massage therapy?

Yes

No

Please explain:(2) What is the role of the CMT in supporting research within the profession of MT?

(3) Are you satisfied with the current regulation of massage therapy research curricula in schools?

Extremely satisfied

Somewhat satisfied

Not sure

Somewhat unsatisfied

Extremely unsatisfied

Please explain: __________________________________________________________

_______________________________________________________________________

(4a) What steps should be taken to improve access to existing research on massage therapy?

________________________________________________________________________________________________________________________________________________________________________________________________________________________

(4b) What steps should be taken to improve research literacy within the community of RMTs in BC?

________________________________________________________________________________________________________________________________________________________________________________________________________________________________________________________________________________________________

(5) In your opinion, what aspects of massage therapy require further research? For example:

Safety

Efficacy

Cost-effectiveness

Other (please specify).

(6) In your view, how may the MT profession move forward?

_______________________________________________________________________________________________________________________________________________

(7) What are the biggest challenges facing the regulation of RMTs in BC?

________________________________________________________________________________________________________________________________________________________________________________________________________________________

(8) Would the CMT be supportive of movements towards gaining a university degree for RMTs at a public university?

YES

NO

Please explain:_________________________________________________________________________________________________________________________________________________________________________________________________________________

(9) Do you have any other comments about the regulation of massage therapy?

________________________________________________________________________________________________________________________________________________________________________________________________________________________

(10) Is the CMT interested in participating in cooperative research projects with the MTA and/or possibly other organizations?

Yes

No

Please explain:

________________________________________________________________________________________________________________________________________________________________________________________________________________________________________________________________________________________________

## Insurance Company Questionnaire

1 a) What are the top 5 health issues that you deal with?

b) Do you believe that RMTs (Registered Massage Therapists) effectively address any of these issues?

Yes—please provide a detailed explanation for each one--above, next to the issues you listed in 1a

No

2 a) What are your views about registered massage therapists in BC?

b) Positive

Somewhat positive

Not sure

Negative

(3)Do you think that massage therapy provides positive health benefits?
Yes—please specify ie what types of health benefits?

Somewhat—please explain ie in which situations?

Not sure—please explain:

No—please explain:

4a Do you set limits to billing by massage therapists?

Always—please explain why

Sometimes—please explain why

Rarely—please explain

Never—please explain

b) What are the billing ranges that your insurance company sets for MT (overall) per year?

5a) Do you have rules about the number of times massage therapy sessions can be provided to patients per year?

No limits

More than 10 visits per patient

3-10 visits per patient

Less than 3 visits per patient

b) Does your company set a limit on the length of each MT session?

Yes—how many minutes:

No

(6) Do you restrict massage therapists to particular types of MT they can provide?

No

YES: Please specify type of massage therapy e.g. Swedish massage, myofascial release

Depends: Please explain

7a) Do you utilize research literature in the work you do?

Yes—which source do you utilize please?

Somewhat

Not sure

No

b) If we were to send you research literature on MT what would you want and in what format please?
Type of research:

Format:

Length:

(8)What would it take for your company to cover Registered massage Therapists more widely for more visits and larger maximum annual pay outs?

Research—evidence of effectiveness

Legislation

More endorsement from MDs

Proof of cost effectiveness

Not sure

Others

(9)In your view how could massage therapy improve its status within the health care field in BC?

10a) What are the main barriers to increased massage therapy coverage in your view?

b) Please rank from 1 to 6 with 1 being the biggest barrier and 6 being the lowest.

Lack of research on efficacy

Lack of fiscal resources (money)

Lack of knowledge about massage therapy ie the active therapeutic component

Competition among providers such as chiropractors and physiotherapists

Lack of widespread understanding of MTs’ full scope of practice

Other: please explain:

(11)Do you believe that massage therapy successfully prevents disease and treats injury?

Yes—please explain:

Somewhat—please explain:

Not sure—please explain:

No—please explain:

12a What kind of research would make Registered Massage Therapists more appealing to you company?

b)Please rank the kind of research that would make MT more appealing to your company (from 1-4 with 1 being the most important and 4 being the least important)

Randomized controlled trials

Cohort and case control studies

Descriptive studies

Other: Please specify____________________________________________

(13) Is there anything else you would like to recommend to the Massage Therapists’ Association about advancing the profession of Massage Therapy, or about the role of research in doing so?

## Massage Therapy Schools Questionnaire

| 1.a | **Please describe the research courses/content that your school offers i.e. in which year, for how long, the content, the expertise of the faculty member.** |
| --- | --- |
| 1.b | **How is this education on research delivered? Please read the list below and indicate how and when the education took place by checking the appropriate education delivery category.**   1. Workshop or seminar 2. Lecture(s) 3. Structured clinical experience 4. Part of one or more PBL (problem-based learning) cases 5. Independent study or self-directed experience 6. Small group discussion or presentation 7. Other: |
| 1.c | **Is your school’s research education on massage therapy:**   1. Part of a separate required course 2. Available as an elective or selective 3. Presented as student-developed lectures or presentations 4. Integrated into many courses throughout the curriculum 5. Other_____ |
| 2. | **Would you say that the majority of your graduates/students are enthusiastic about research?**  Yes (please explain)  No (please explain)  Depends (please explain) |
| 3.a | **How much do graduates from your program know about the effectiveness of massage therapy from an evidence-based perspective?**   1. Well informed 2. Substantial knowledge 3. Somewhat informed 4. Poorly informed |
| 3.b | **Do your students/graduates consult peer-reviewed literature regularly?**  Yes (please explain)  No (please explain)  Depends (please explain) |
| 3.c. | **Can the majority of your graduates design and conduct independent research?**   1. Strongly agree 2. Somewhat agree 3. Not sure 4. Somewhat disagree 5. Strongly disagree |
| 3.d. | **How would you rank the ability of your graduating students to evaluate the strengths, weaknesses, and appropriate applications of a range of research methodologies to the area of massage therapy?**   1. Excellent 2. Very good 3. Good 4. Fair 5. Poor |
| 4 | **In your view, how important is research knowledge to the massage therapy profession?**   1. Very important 2. Moderately important 3. Somewhat important 4. Not important |
| 5. | **How would you rate faculty members’ willingness to engage in research?**   1. High 2. Moderate 3. Mild 4. Low |
| 6.a | **What are the barriers to massage therapy research in your school?** |
| 6.b. | **Please rank, in order of importance, these barriers, with 1 as the biggest barrier.**   1. Restricted curriculum space 1 2. Faculty members' limited (paid) time 3. The explosion of information in complementary and alternative medicine 4. Great variation in approach 5. Content and depth of the programs 6. Lack of Funding 7. Lack of Interest/Expertise in Research 8. Others__________________________________________________________ |
| 7.a | **How can these barriers be addressed?** |
| 7.b. | **Please rank initiatives to stimulate greater research content, with 1 being the most important initiative:**   1. Greater consensus on the ingredients of a core curriculum on research and its goals 2. Training for faculty 3. Further incentives for research literacy to be part of the curriculum 4. Funding initiatives 5. Attracting more researchers to schools who train therapists to be researchers 6. Collaborating with the MTABC and CMTBC to develop, test and evaluate a best practice guideline as a way of contributing to putting research into practice and mentoring students to carry out research 7. Other: please specify |
| 8. | **In your view, what steps do RMTs need to take in order to elevate the status of the profession?** |
| 9.a | **Please suggest ways in which various institutions within the profession (the College, the MTA, schools etc) could work together to encourage research literacy and activity:** |
| 9.b. | **Would you/your school be interested in collaborating with others such as the MTABC and CMTBC to develop, test and evaluate a best practice guideline to mentor students to carry out research?** Yes  No |
| 10. a | **Are the research competencies in the Occupational Competency Profile relevant to the students, clinical instructors, academic instructors, executive etc?**  Yes (please explain)  No (please explain) |
| 10.b. | **Are the competencies:**  integrated throughout the curriculum?  just in one course? |
| 10.c. | **What would it take to integrate research throughout the curriculum?**  i) What support would you need to be able to integrate the competencies throughout the curriculum i.e. what would facilitate integration of research competencies into your curriculum? |
| 11. | **Is there anything you wish to add?** |
